# Supplementary material for: Migration confers winter survival benefits in a partially migratory songbird
Source: eLife. 2017 Nov 21;6:e28123. doi: 10.7554/eLife.28123 (PMC5697929; doi:10.7554/eLife.28123)
Supplement: Transparent reporting form [file elife-28123-transrepform.docx]

***eLife’s* transparent reporting form**

We encourage authors to provide detailed information *within their submission* to facilitate the interpretation and replication of experiments. If you have any questions, please contact us: [editorial@elifesciences.org](mailto:editorial@elifesciences.org).

**Sample-size estimation**

- You should state whether an appropriate sample size was computed when the study was being designed
- You should state the statistical method of sample size computation and any required assumptions
- If no explicit power analysis was used, you should describe how you decided what sample (replicate) size (number) to use

Please outline where this information can be found within the submission (e.g., page numbers or figure legends), or explain why this information doesn’t apply to your submission:

We did not compute a sample size, in this field study we aimed to catch 100 birds per year (7 years of study). We settled on this number based on radio tags availability. In MS lines 255-271, we report the effective sample size (262 birds: 192 residents, 70 migrants).

**Replicates**

- You should report how often each experiment was performed
- You should include a definition of biological versus technical replication
- The data obtained should be provided and sufficient information should be provided to indicate the number of independent biological and/or technical replicates
- If you encountered any outliers, you should describe how these were handled
- Criteria for exclusion/inclusion of data should be clearly stated
- High-throughput sequence data should be uploaded before submission, with a private link for reviewers provided (these are available from both GEO and ArrayExpress)

Please outline where this information can be found within the submission (e.g., page numbers or figure legends), or explain why this information doesn’t apply to your submission:

In the technical sense our experiment was replicated only once (since we studied only one population). Data preparation and handling details are provided in MS lines 272-284

**Statistical reporting**

- Statistical analysis methods should be described and justified
- Raw data should be presented in figures whenever informative to do so (typically when N per group is less than 10)
- For each experiment, you should identify the statistical tests used, exact values of N, definitions of center, methods of multiple test correction, and dispersion and precision measures (e.g., mean, median, SD, SEM, confidence intervals; and, for the major substantive results, a measure of effect size (e.g., Pearson's r, Cohen's d)
- Report exact p-values wherever possible alongside the summary statistics and 95% confidence intervals. These should be reported for all key questions and not only when the p-value is less than 0.05.

We report the statistical test, description and justification in ms lines 285 - 300. Because we used an information-theoretic approach details of the different models we tested are found in the results table 1.

Effective Sample size was reported ms lines 255-271

We report, mean and confidence intervals for the results lines 92-121. We also report AIC values used for the model selection in table 1 MS lines 445 Fig 3 presenting the main result also contain mean and 95% confidence intervals.

Please outline where this information can be found within the submission (e.g., page numbers or figure legends), or explain why this information doesn’t apply to your submission:

(For large datasets, or papers with a very large number of statistical tests, you may upload a single table file with tests, Ns, etc., with reference to page numbers in the manuscript.)

**Additional data files (“source data”)**

- We encourage you to upload relevant additional data files, such as numerical data that are represented as a graph in a figure, or as a summary table
- Where provided, these should be in the most useful format, and they can be uploaded as “Source data” files linked to a main figure or table
- Include model definition files including the full list of parameters used
- Include code used for data analysis (e.g., R, MatLab)
- Avoid stating that data files are “available upon request”

Please indicate the figures or tables for which source data files have been provided:

Source data files have been provided for figure 2 panel a and b, figure 3 and the data source for table 1. Full model definition and results are provided in the file called EsurgeSession.zip (see readme on the file for more details)
